# Supplementary material for: Mitochondrial Ceramide Effects on the Retinal Pigment Epithelium in Diabetes
Source: Int J Mol Sci. 2020 May 28;21(11):3830. doi: 10.3390/ijms21113830 (PMC7312467; doi:10.3390/ijms21113830)
Supplement: Supplementary file 1 [file ijms-21-03830-s001.pdf]

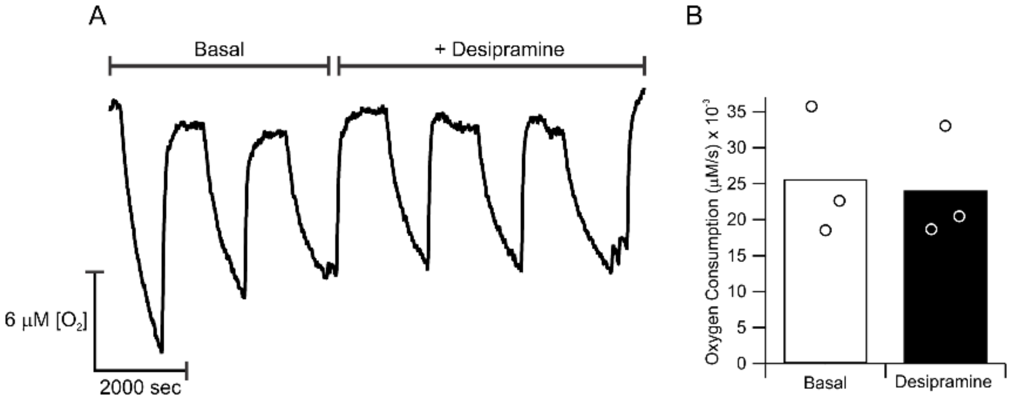

Figure 1. Microrespirometry of bovine retinal endothelial cells (BREC). (**A**) Oxygen concentration trace of BREC substrate-supported respiration (basal) and substrates + 15 μM desipramine delivered on-chip (desipramine). (**B**) Substrate-supported oxygen consumption rates of BREC with or without 15 μM desipramine.

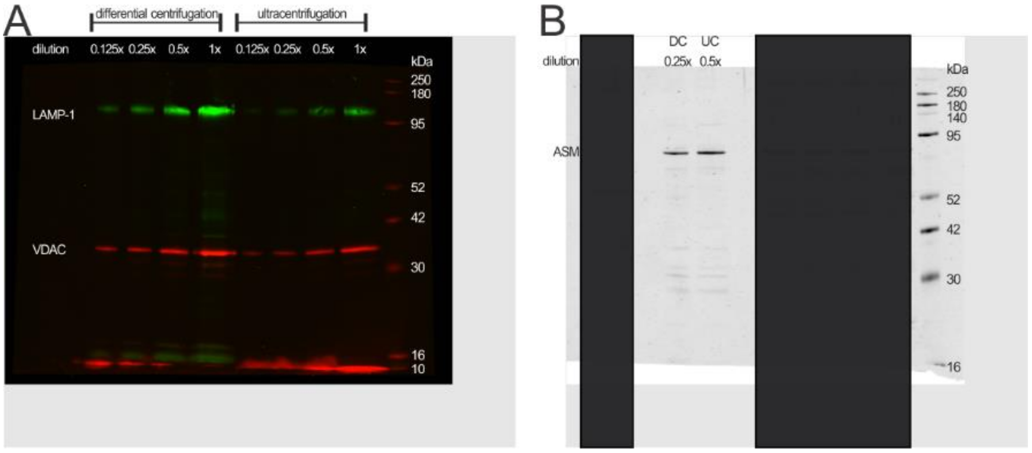

Figure 2. Immunoblots of lysosomal and mitochondrial markers (**A**) and ASM (**B**) at varying levels of mitochondrial purity. LAMP-1 = lysosome associated membrane protein 1, VDAC = voltage dependent cation channel, ASM = acid sphingomyelinase, black boxes = unrelated experiment.

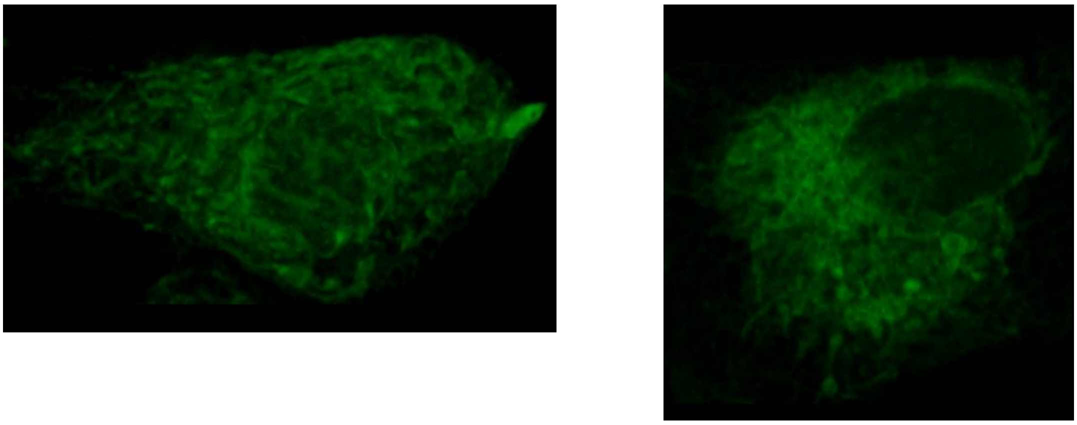

Figure 3. 3D reconstructed animation of control-derived (left) and diabetic-derived (right) RPE cells. Cells were stained with MitoTracker Green, imaged with confocal microscopy and reconstructed using ImageJ. See main text for details.
